# Supplementary figures and images for: Evaluating land-cover change and land subsidence in coal fire zones: Insights from multi-source monitoring
Source: PLoS One. 2025 May 28;20(5):e0322284. doi: 10.1371/journal.pone.0322284 (PMC12118845; doi:10.1371/journal.pone.0322284)

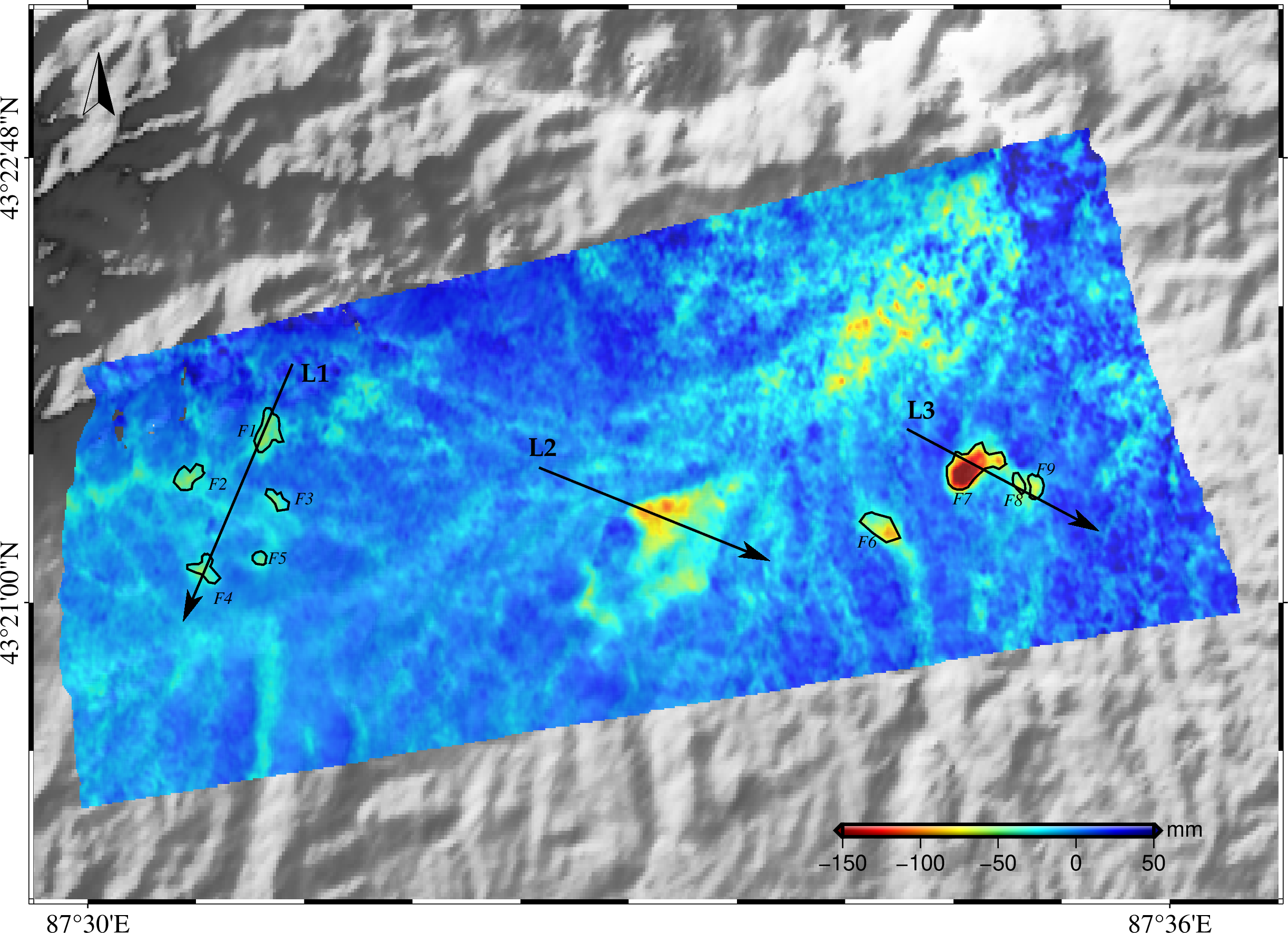

Supplement: Source data — (ZIP) [file pone.0322284.s001.zip › Source data/Fig8/A1.png]

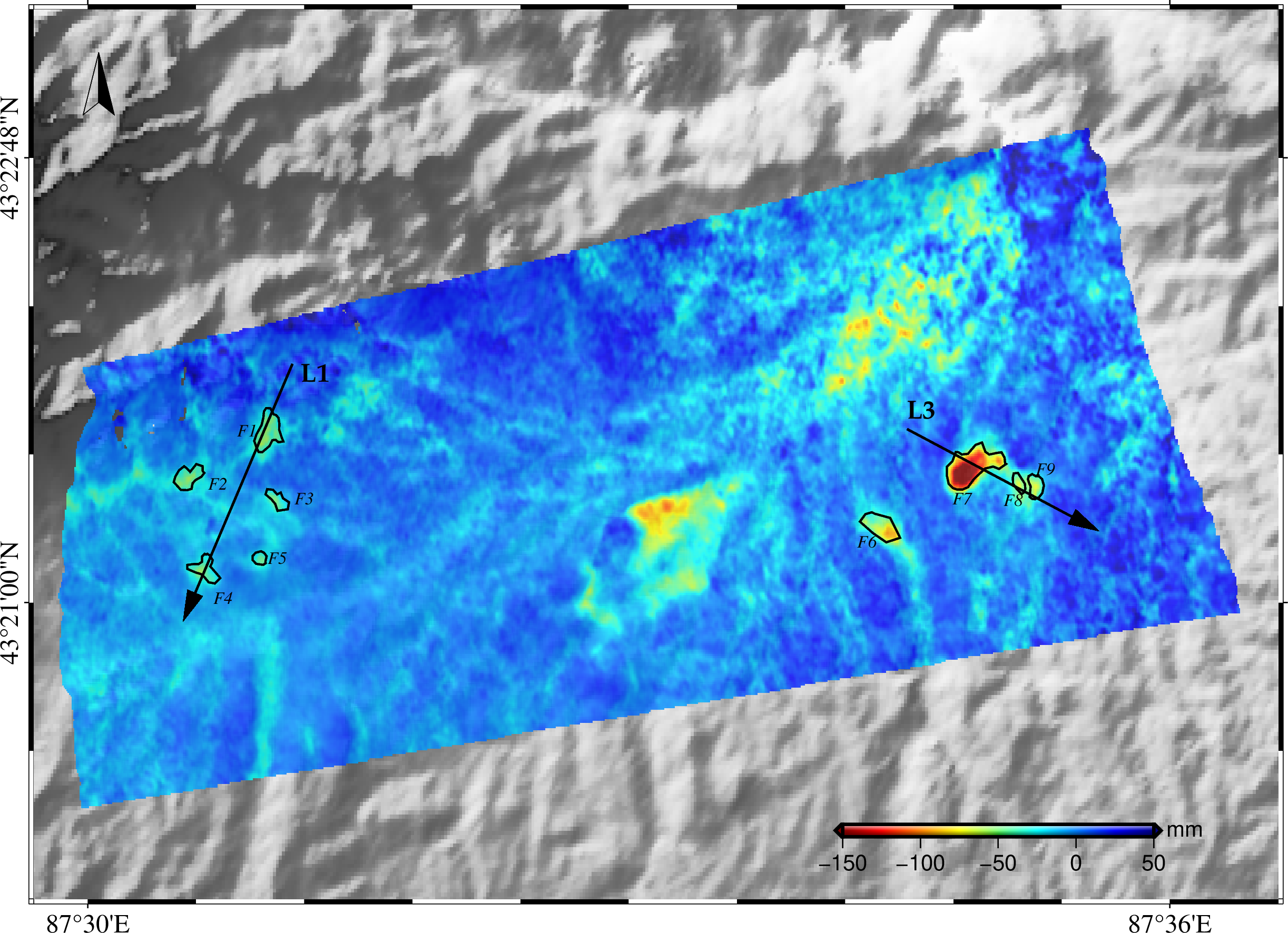

Supplement: Source data — (ZIP) [file pone.0322284.s001.zip › Source data/Fig8/A2.png]

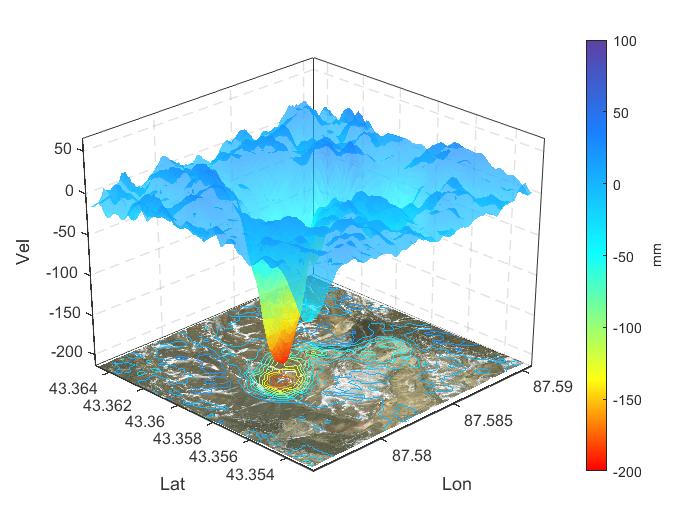

Supplement: Source data — (ZIP) [file pone.0322284.s001.zip › Source data/Fig8/B.jpg]

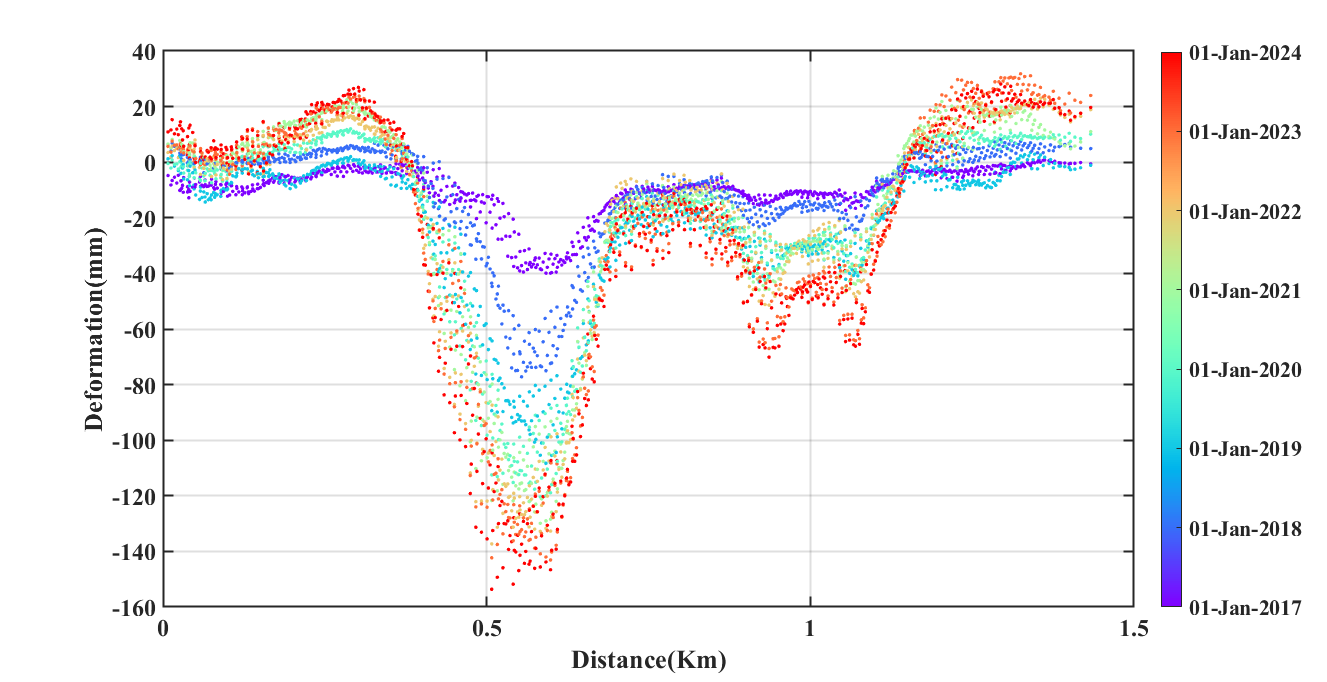

Supplement: Source data — (ZIP) [file pone.0322284.s001.zip › Source data/Fig8/c.bmp]

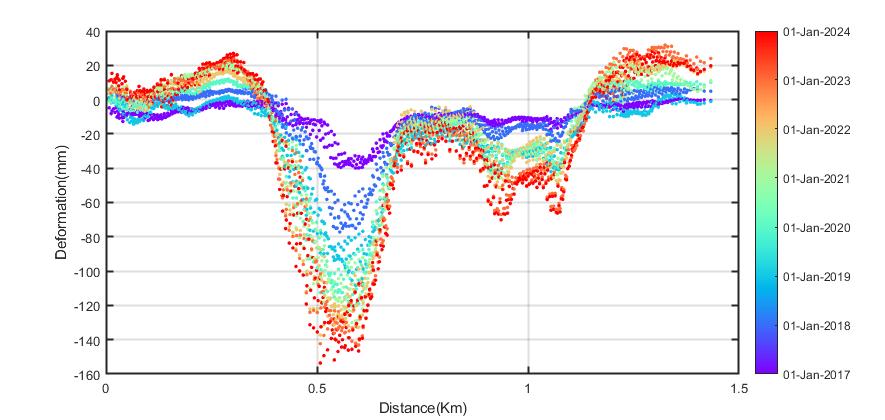

Supplement: Source data — (ZIP) [file pone.0322284.s001.zip › Source data/Fig8/c.jpg]

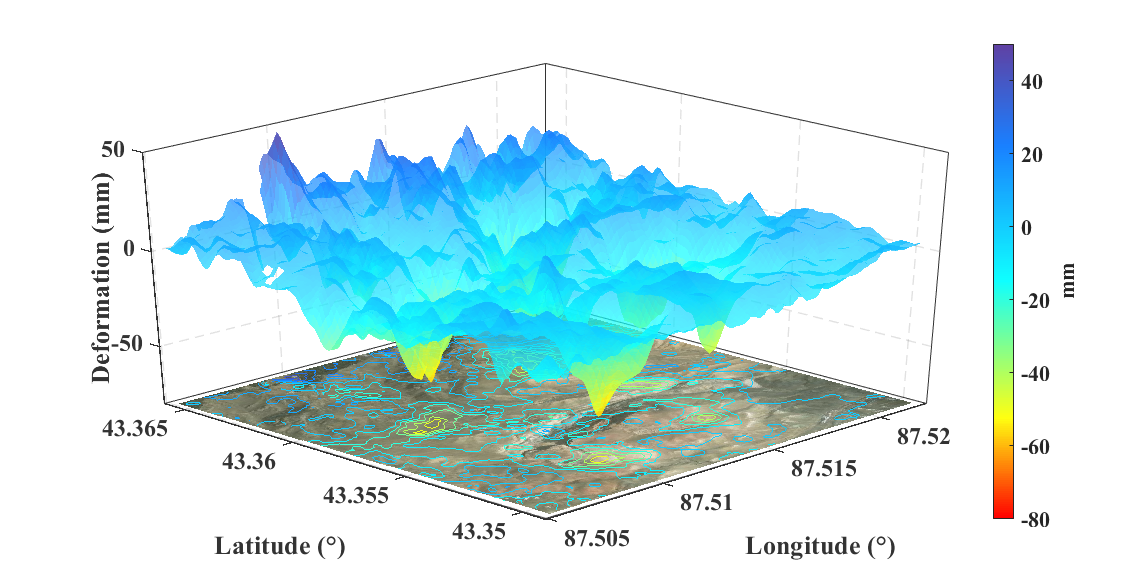

Supplement: Source data — (ZIP) [file pone.0322284.s001.zip › Source data/Fig8/d.bmp]

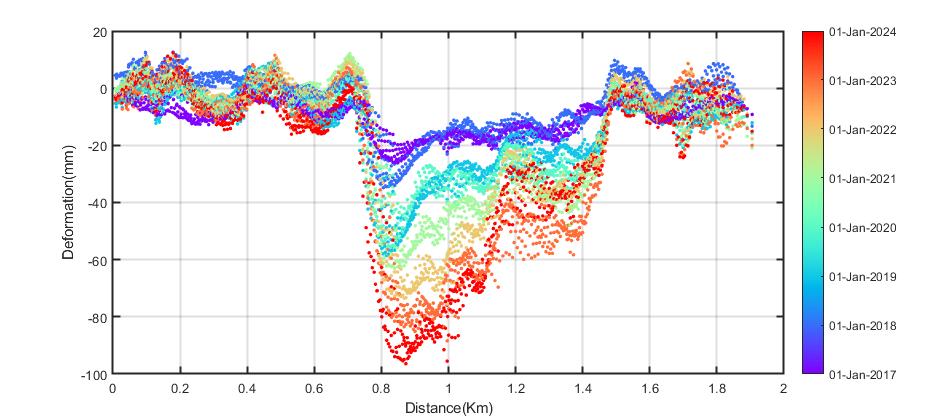

Supplement: Source data — (ZIP) [file pone.0322284.s001.zip › Source data/Fig8/d.jpg]

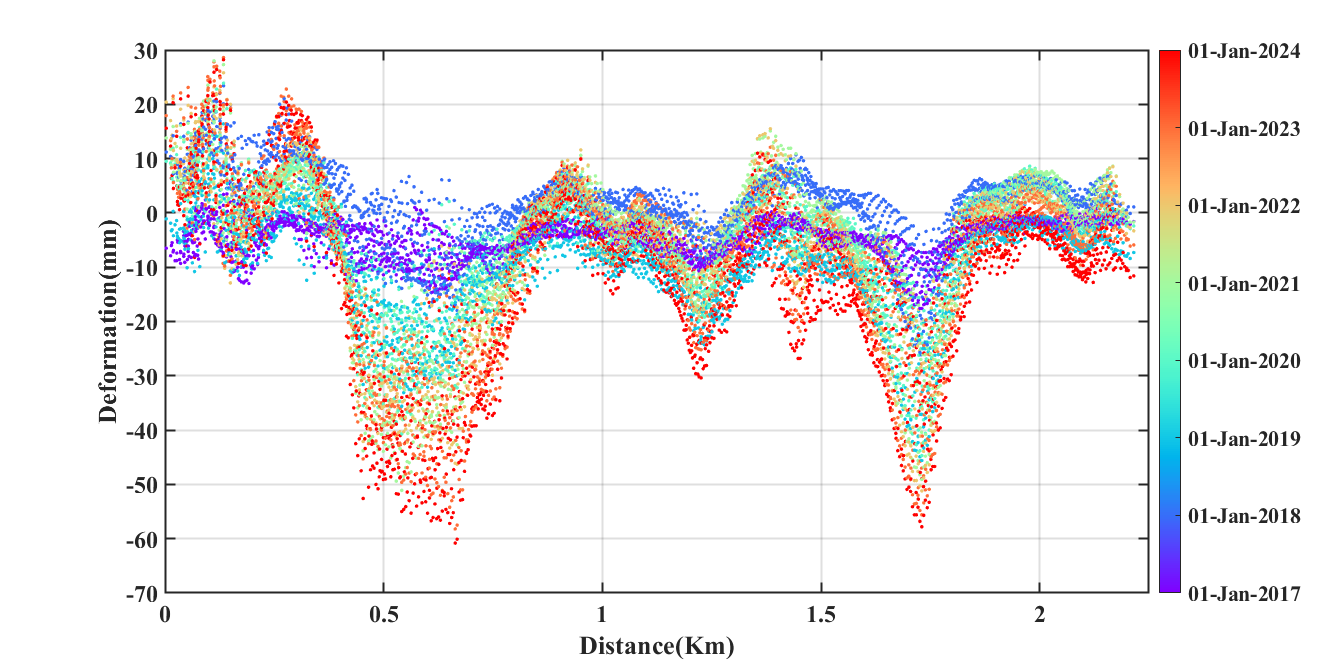

Supplement: Source data — (ZIP) [file pone.0322284.s001.zip › Source data/Fig8/e.bmp]

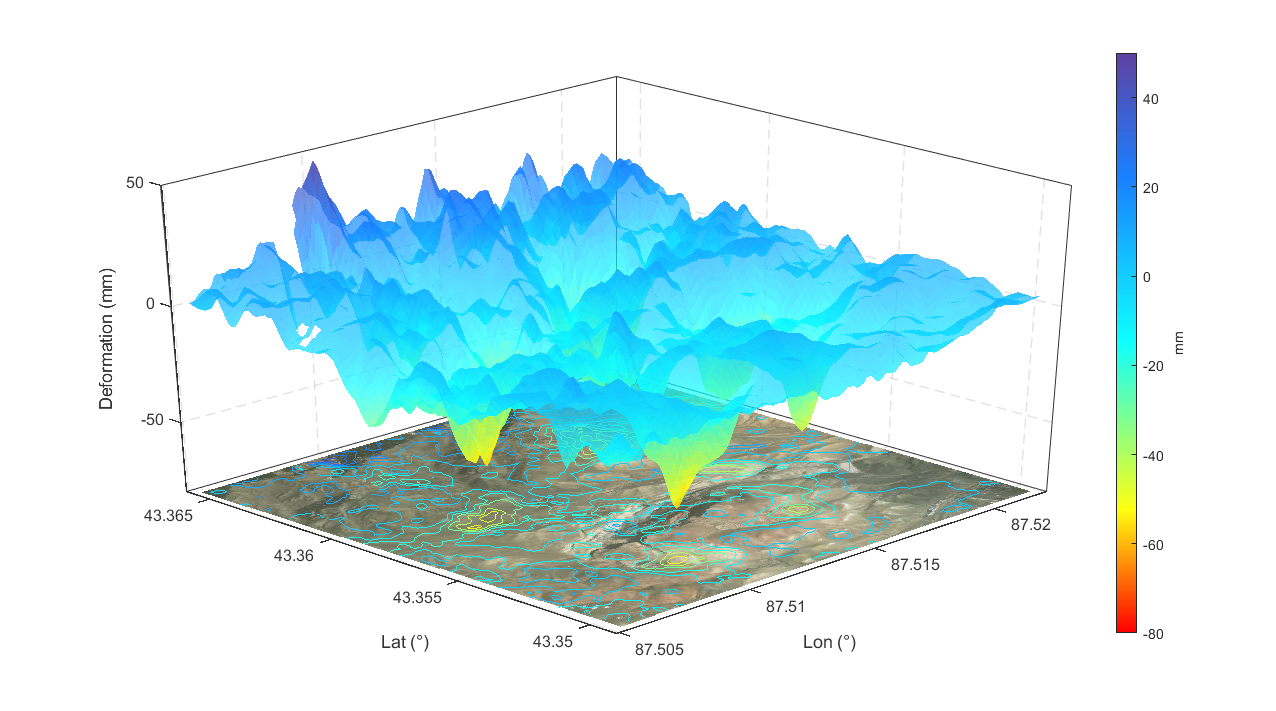

Supplement: Source data — (ZIP) [file pone.0322284.s001.zip › Source data/Fig8/f.bmp]

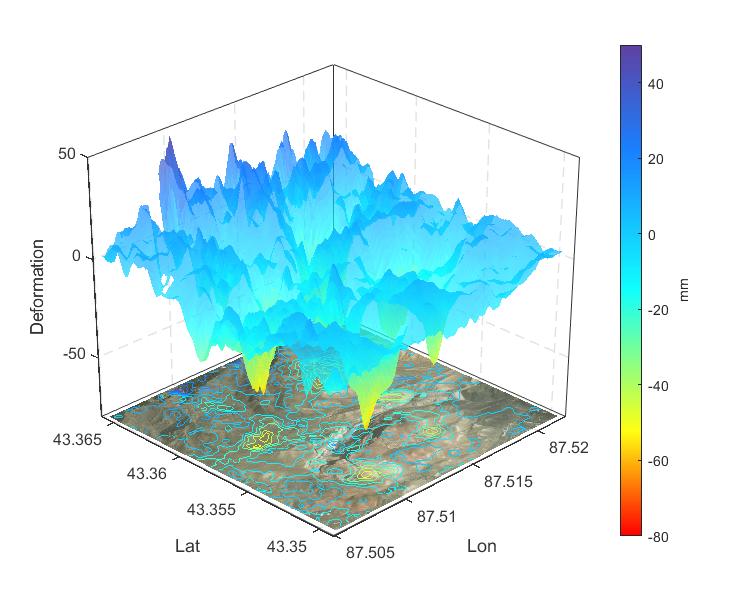

Supplement: Source data — (ZIP) [file pone.0322284.s001.zip › Source data/Fig8/f.jpg]
